# Development and Validation of an On-Line Water Toxicity Sensor with Immobilized Luminescent Bacteria for On-Line Surface Water Monitoring

**DOI:** 10.3390/s17112682

**Published:** 2017-11-22

**Authors:** Marjolijn Woutersen, Bram van der Gaag, Afua Abrafi Boakye, Jan Mink, Robert S. Marks, Arco J. Wagenvoort, Henk A. M. Ketelaars, Bram Brouwer, Minne B. Heringa

**Affiliations:** 1 National Institute for Public Health and the Environment (RIVM), P.O. Box 1, 3720 BA Bilthoven, The Netherlands; minne.heringa@rivm.nl; 2 PAREXEL International, The Quays, 101-105 Oxford Rd, Uxbridge UB8 1LZ, UK; abrafi_boakye@live.co.uk; 3 VTEC Lasers & Sensors, Kastanjelaan 400, 5616 LZ Eindhoven, The Netherlands; janmink2005@gmail.com; 4 Department of Biotechnology Engineering, Faculty of Engineering Science, Ben-Gurion University of the Negev, Beer-Sheva 84105, Israel; rsmarks@exchange.bgu.ac.il; 5 National Institute for Biotechnology in the Negev, Ben-Gurion University of the Negev, Beer-Sheva 84105, Israel; 6 The Ilse Katz Center for Meso and Nanoscale Science and Technology, Ben-Gurion University of the 6egev, Beer-Sheva 84105, Israel; 7 AqWa ecologisch advies, Voorstad 45, 4461 KL Goes, The Netherlands; aqwa@zeelandnet.nl; 8 Evides Water Company, Schaardijk 150, 3063 NH Rotterdam, The Netherlands; h.ketelaars@evides.nl; 9 Vrije Universiteit Faculty of Earth & Life Sciences, Department of Animal Ecology, De Boelelaan 1085, 1081 HV Amsterdam, The Netherlands; bram.brouwer@bds.nl; 10 BioDectection Systems, Science Park 406, 1089 XH Amsterdam, The Netherlands

**Keywords:** biosensor, bacteria, luminescence, genotoxicity, water monitoring

## Abstract

Surface water used for drinking water production is frequently monitored in The Netherlands using whole organism biomonitors, with for example *Daphnia magna* or *Dreissena* mussels, which respond to changes in the water quality. However, not all human-relevant toxic compounds can be detected by these biomonitors. Therefore, a new on-line biosensor has been developed, containing immobilized genetically modified bacteria, which respond to genotoxicity in the water by emitting luminescence. The performance of this sensor was tested under laboratory conditions, as well as under field conditions at a monitoring station along the river Meuse in The Netherlands. The sensor was robust and easy to clean, with inert materials, temperature control and nutrient feed for the reporter organisms. The bacteria were immobilized in sol-gel on either an optical fiber or a glass slide and then continuously exposed to water. Since the glass slide was more sensitive and robust, only this setup was used in the field. The sensor responded to spikes of genotoxic compounds in the water with a minimal detectable concentration of 0.01 mg/L mitomycin C in the laboratory and 0.1 mg/L mitomycin C in the field. With further optimization, which should include a reduction in daily maintenance, the sensor has the potential to become a useful addition to the currently available biomonitors.

## 1. Introduction

Contamination of surface water with various pollutants is a well-known worldwide problem, especially in developing countries [1,2,3]. It poses a particular challenge for the drinking water companies that use surface water as a source for drinking water [4]. Accidents with ships, runoffs from fields or discharges from factories can cause sudden peaks in the concentrations of pollutants in rivers [5]. To prevent the intake of these plumes of contaminants, the water quality is monitored upstream of the drinking water inlet points in some countries, including The Netherlands. The devices that are in use for on-line monitoring can be divided in three categories. The first are sensors that measure chemical or physical parameters, such as temperature, conductivity, turbidity, acidity, metal ions [6,7,8], or oxygen content. The second group measures several specific organic pollutants by chemical analysis such as on-line HPLC-UV. The third group can be sub-divided into living biological systems, for example biomonitors with *Daphnia*, algae, or *Dreissena* mussels, which respond to the toxicants in the water [9,10,11] and biosensors, which employ natural or artificial bio-recognition elements anchored to a surface that respond to the toxicants in the water [12]. If the concentration of a pollutant or the response of a biomonitor reaches a certain critical value, an alarm is generated and the water intake is closed. 

However, not all pollutants that can have adverse effects in humans are detected by the panel of current biomonitors or on-line chemical analysis instruments. Especially compounds that are not detected by UV (due to the lack of a conjugated system in the molecule), or do not affect the organisms used in the biomonitors, or only cause long term effects in low concentrations are easily missed. Therefore, there is a demand for on-line biosensors that can detect these human-relevant toxic compounds and mixtures in water in real-time. 

In this study, such an on-line biosensor has been developed by using genetically modified bacteria in which a promoter gene for a specific stress response is coupled to genes for luciferase (*lux*). Although several luminescent bacteria are available for use in this device, these experiments were all performed with an *Escherichia coli* strain DPD2794, which contains the *luxCDABE* genes for luciferase coupled to the *recA* promoter gene from the SOS response, which is activated by DNA damage [13]. This strain was found to be the most relevant of the available strains, for the purpose of surface water inlet monitoring for drinking water production [14]. 

Various configurations of such bioreporter-based biosensors have been built and tested, such as miniature bioreactors with bacteria in suspension [15,16], a sensor with freeze-dried bacteria [17], biochips [18], a sensor with bacteria immobilized in a disposable card [19], and self-contained sensors with bacteria immobilized onto optical fiber tips [20,21,22], later adapted as a flow-through sensor system [22]. The latter was promising, since it was one of the systems that could be used on-line and the bacteria in the sensor were relatively easy to change. However, this sensor was not yet capable of long term measurements while exhibiting limitations in sensitivity and sturdiness. For this reason, the current study was started in which a new prototype and improved version was built with some major adjustments. A dual immobilization system was integrated enabling the comparison of a larger glass slide conformation with that of the fibre optic. As only a small portion of the emitted light reaches the photomultiplier through the fiber [20], it was expected that the capture of the emitted light of a larger surface would increase the sensitivity. 

In addition, new features were integrated, such as control over temperature, flow speed, concentration of growth medium, and the addition of positive controls. The instrument was built with inert materials, to prevent biofouling, leaching of toxic compounds, loss of spiked compounds, or memory effects from the sensor or tubing. In order to ensure no living genetically modified bacteria would be released into the environment (due to replication of the bacteria), the efflux water was sterilized with chlorine. For the field study, an UV-C unit was installed behind the sensor for on-line disinfection of the wastewater, as an alternative to the chlorination of collected batches of wastewater.

The new sensor prototype was constructed and first tested in the laboratory to determine the response characteristics. Eventually the sensor was transported to the field monitoring station Keizersveer (near Hank) along the river Meuse in The Netherlands and tested on site for a period of two months. During this time, the background signal was determined and several spike experiments were performed. The signal of the sensor was compared with the alarms of the *Daphnia*toximeter (bbe-Moldaenke, Kiel, Germany) and Musselmonitor^®^ (AquaDect, Brouwershaven, The Netherlands) at the same station, and with the measurements of the chemical and physical sensors that were active during this period.

## 2. Materials and Methods

### 2.1. Chemicals

Mitomycin C, ampicillin, and tetramethyl orthosilicate (TMOS) were purchased from Sigma Aldrich (Zwijndrecht, The Netherlands). Nalidixic acid, lysogeny broth (LB) powder, agar, dimethyl sulfoxide, acetone, glycerol, NaCl, KCl, NaHPO_4_·2H_2_O, KH_2_PO_4_, HCl, and NaOH were purchased from Boom (Meppel, The Netherlands). All chemicals were of analytical grade and stored as suggested by the manufacturer’s instructions. 

### 2.2. Bacteria and Growth Conditions

The bacterial strain used in the sensor was DPD2794 (kindly donated by Prof. S. Belkin through Prof. Marks, Ben Gurion University, Beer Sheva, Israel)). DPD2794 is a genetically modified *Escherichia coli* strain, which contains a plasmid with the *recA* promoter, coupled to the *luxCDABE* genes. The *recA* gene is part of the bacterial SOS response, which generates DNA damage repair [13]. The *luxCDABE* genes are derived from *Aliivibrio fischeri* and code for the enzyme luciferase and the synthesis of its substrate. As a result of the coupling of these genes, DPD2794 generates light with a peak wavelength of 490 nm when DNA damage is induced [23], without the need of adding a substrate. A characterization of the response of this strain to various compounds performed in 96-wells plates can be found in the Supplementary data, Part I. This characterization was performed without metabolic activation (S9-mix), as this cannot be used in the sensor. Stock cultures of bacteria were stored at −80 °C in 25% (v/v) glycerol medium. Colonies for daily use were maintained at 4 °C on LB-agar plates, supplemented with 100 mg/L ampicillin. Before each experiment, bacteria were grown overnight in an incubator at 26 °C (Friocell, MMM, Planegg, Germany). Bacteria were grown in LB broth which contained 100 mg/L ampicillin. The cultures were diluted to an OD_600_ of 0.2 as determined by a spectrophotometer (Jenway 6300 VIS, Staffordshire, UK). 

### 2.3. Immobilization in the Sensor

The sol-gel was prepared by mixing 4 mL TMOS, 2.4 mL ultrapure water, and 0.68 mL of 0.1 M HCl. This mixture was left for 24 h at 4 °C to pre-polymerize. The glass slides were standard microscope slides (Waldemar Knittel, Braunschweig, Germany) purchased from Boom. The optical fibers used were HUV-H pigtail fibers (Ceramoptec, Bonn, Germany). They had a core of pure, synthetic, fused silica with a diameter of 1800 µm and a numerical aperture of 0.48 ± 0.2. The cladding was of hardpolymer with a diameter of 1900 µm. The tip on one end was polished; on the other end a SMA-standard connector was attached. 

**Figure 1:**
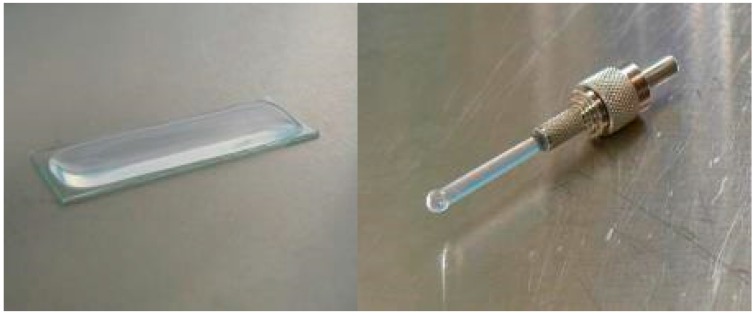
The bacteria are immobilized on the tip of an optical fiber and a microscope slide.

The pre-polymerized gel was first mixed with 0.05 M NaOH and then immediately mixed with the bacterial culture in a ratio of 1:1:4 sol-gel:NaOH:bacteria. One mL of the sol-bacteria mixture was immediately brought on the surface of the glass slide in a rectangular area of approximately 10 cm^2^ and with a thickness around 1 mm. A drop with a height of approximately 3 mm was attached to the tip of the fiber by keeping the fiber upside down and letting the drop flow to the tip from a pipette (Figure 1). Both slide and fiber were left for 5 min to let the gel polymerize. 

### 2.4. System Setup

**Figure 2:**
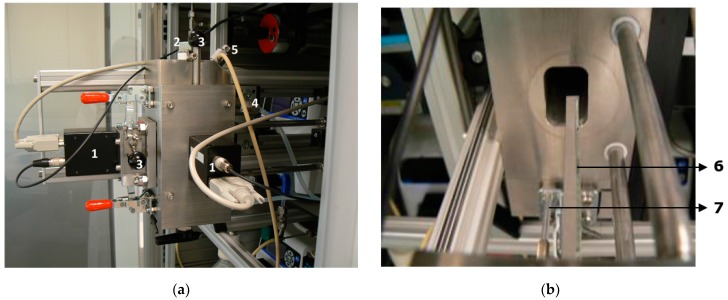
Photographs of the measurement chamber as seen from the outside (**a**) and from the inside (**b**) of the pulled-out chamber side. The numbers correspond with the following parts: 1. photomultiplier tubes, 2. pH and temperature sensor inlet, 3. light shutters, 4. water outlet, 5. air outlet, 6. slide, 7. fiber.

The glass slide and optical fiber with the attached sol gel-bacteria mixture were fixed in a stainless steel measurement chamber, see Figure 2.

**Figure 3:**
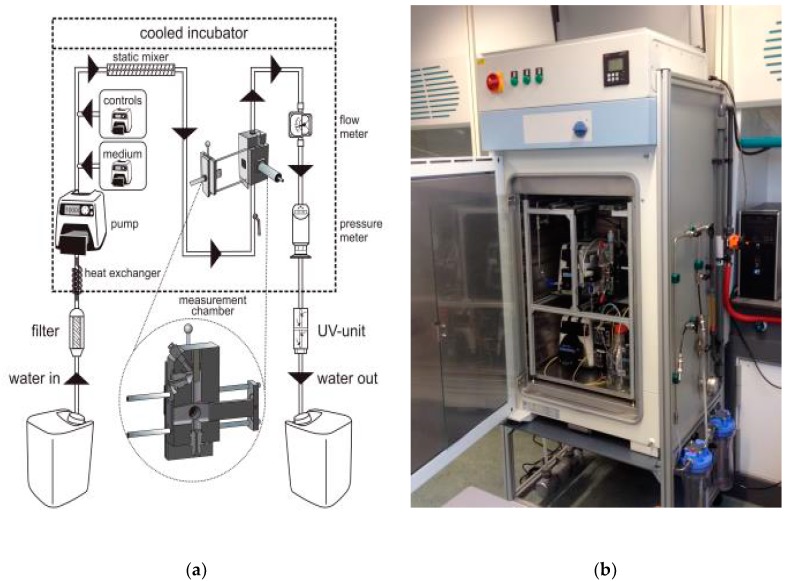
(**a**) A schematic overview of the sensor, the inside of the measurement chamber is shown in the enlargement. (**b**) Photograph of the sensor at monitoring station Keizersveer. The filters were attached to the side of the sensor (on the right). The UV-unit is visible on the bottom left.

The side of the chamber was easily removable to enable efficient changing of the probes (Figure 2 and Figure 3). This chamber was designed by 2M Sensors (Eindhoven, The Netherlands) and the assemblage was done at KWR Watercycle Research Institute (Nieuwegein, The Netherlands). The inner volume of the chamber was reduced as much as possible, down to a volume of 65 mL. This was done to prevent a reduction or delay of the response by dilution of a plume of compounds in the chamber. The inner corners of the chamber were rounded both to reduce the volume and to ease the removal of biofilm. Tap water flowed from bottom to top through the chamber, which was equipped with an air outlet at the top and a tap at the bottom to be able to drain the water. Two photomultiplier tubes (H-7467, Hamamatsu Photonics, Shimokanzo, Japan) were connected to measure both the slide and fiber light signal outputs separately. The fiber was placed behind the slide holder so both were shielded from each other’s light. Both photomultipliers were protected from overstimulation (e.g., when the chamber was opened to change the slide and fiber) by manually operated light shutters. The luminescence was measured four times per minute with an integration time of 10 milliseconds during the laboratory experiments and 100 ms during the field tests. All data points presented are averages over five min.

An overview of the entire sensor is provided in Figure 3. During experiments the total water flow through the sensor was maintained at 25 mL/min. Before the water entered the chamber, LB medium was added by an independent pump. The concentration LB medium was maintained at 2% in the laboratory and 0.2% in the field, as this gave the optimal response (Supplementary data, Part II).

To be able to verify the responsiveness of the bacteria and to characterize the system, compounds could be added at will through a third independent pump. Nalidixic acid and mitomycin C were used as positive controls and added to the water after the medium. Exposure to positive controls was always for a period of one h. Mitomycin C was used as a positive control when it was necessary to use the most potent genotoxin (e.g., for sensitivity determination). In all other cases, nalidixic acid was used as this compound was less hazardous, facilitating working conditions and waste removal. To ensure an even distribution of these additions, a static mixer (SS tube mixer, Cole-Parmer, Vernon Hills, IL, USA) was installed before the measurement chamber.

As an extra safety measure, the water passed through a flow meter (M-21, Tecfluid, Sant Just Desvern, Spain) and pressure meter (Ceraphant, Endress+Hauser, Reinach, Switzerland) after the measurement chamber. All tubing in the system, apart from the dosing of LB, was of either stainless steel or Teflon to prevent loss of compounds and contaminating ‘memory’ effects. Masterflex PharMed tubing (Cole Palmer, Vernon Hills, IL, USA) was used instead of Teflon for the addition of LB broth, as Teflon was too inflexible for this pump for low volumes and the high LB stock concentration would not be influenced by the tubing. All aforementioned parts were placed in a cooled incubator (Friocell 222, MMM, Planegg, Germany) to stabilize the temperature at 26 °C. The water that entered the sensor was first brought to 26 °C with a heat exchanger inside the incubator. The sensor was controlled and the data was collected with Labview software (version 2011, National Instruments, Austin, TX, USA).

To reduce the formation of fouling in the sensor, two filters were installed up-flow of the incubator before the field experiments. The first was a 0.5 µm cartridge filter and the second a membrane filter with a 30 nm polyvinylidene membrane (Compact PVC module with membrane F4385, Norit X-Flow, Enschede, The Netherlands). When the filters were tested in the laboratory, a separate pump (7523-57 L/S Digital Drive, Masterflex, Schiedam, The Netherlands) was used to push the water over the filters, after which it was gathered in a 50L barrel (Graf, Teningen, Germany). 

The water that left the sensor was collected in a waste barrel, where one litre of bleach was added for disinfection (40 g chlorine per litre). In some experiments, the water went through an 18-Watt UV-C water clarifier (AquaCristal Series II, JBL, Neuhofen, Germany) which was tested as an alternative method for disinfection (see Supplementary data, Part III). The clarifier had three obstacles around the UV-lamp to force the water to flow around the lamp, thus reducing the chance that a portion of the water would not be irradiated. It was installed between the sensor and the waste barrel (visible at the bottom of Figure 3b). 

To clean the entire system, the chamber and tubing in the sensor were flushed with household bleach diluted with tap water in a ratio of respectively 3:7 for approximately 15 min every day. Afterwards, the system was thoroughly flushed with tap water for 1 h.

### 2.5. Tap and Surface Water Experiments in the Laboratory

Before the sensor was moved to the field, the performance of the sensor was tested in the laboratory with tap water and river water samples. Tap water was used to optimize the setup of the sensor and determine the limit of detection with relatively minor fluctuations in water quality. River water was also tested in the laboratory to confirm the proper functioning of the bacteria in surface water. The water was collected from the Lekkanaal in Nieuwegein, The Netherlands, which originates from the river Rhine. The water was filtered and tested in the sensor on the same day as it was collected. 

### 2.6. Field Tests

For the field experiments, the sensor was moved to monitoring station Keizersveer in Hank, The Netherlands. This monitoring station is situated along the river Meuse and is managed by the drinking water company Evides. Other monitors that are employed at the station include the *Daphnia*toximeter (bbe-Moldaenke, Schwentinental, Germany) and the Musselmonitor^®^ (AquaDect, Brouwershaven, The Netherlands), containing *Daphnia magna* and the Quagga Mussel, *Dreissena bugensis*, respectively. Both monitors were also running during the test period. The sensor was tested from the 2nd of July, 2012 until the 7th of September, 2012. At the monitoring station, on-line sensors for temperature, oxygen content, turbidity, conductivity and acidity were used for a general water quality assessment. Besides a regular chemical monitoring program, based on the regulation for drinking water, once a week samples are screened for chemical pollutants using GC-MS and HPLC-UV-DAD by the water laboratory, Aqualab Zuid (Werkendam, The Netherlands). The water at the monitoring station was taken directly from the Meuse with a pipe and first went through a 1.0 mm filter (D-74613, Mahle, Flintbek, Germany), before being distributed to the various monitors in the station. Then it was led through the aforementioned 0.5 µm and 0.03 µm filters for the bacterial sensor specifically and subsequently entered the sensor.

Because the preparatory equipment at the monitoring station was limited, the bacteria had to be immobilized at the laboratory located in Nieuwegein and transported to Hank by car. During the transport, the slide with the immobilized bacteria was submerged in phosphate buffered saline (PBS), with an initial temperature of ±4 °C, and placed in a cool box. All slides were used on the same day as they were prepared. The time between the immobilization of the bacteria and their insertion in the sensor was approximately 1.5–2 h. 

## 3. Results and Discussion

### 3.1. Speed and Sensitivity of the Sensor with Tap Water

The sensor was first tested in the laboratory with tap water to determine its speed and sensitivity under controlled conditions. The limit of detection (LOD) was determined with the ISO standard method for the specification and performance tests of new on-line sensors and analyzing equipment for water [24]. 

**Figure 4:**
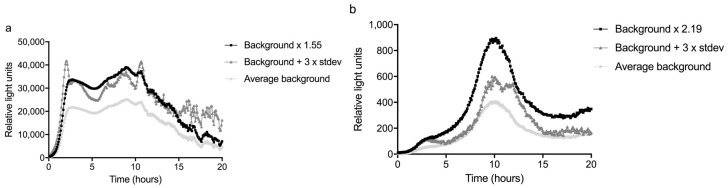
An overview of the average background of three experiments performed in the laboratory with tap water to determine the normal background response of the sensor. Shown are the background + three times standard deviation and the background x the highest response ratio between 2 and 10 h. This ratio was 1.55 for the glass slide (**a**) and 2.19 for the fiber (**b**).

**Figure 5:**
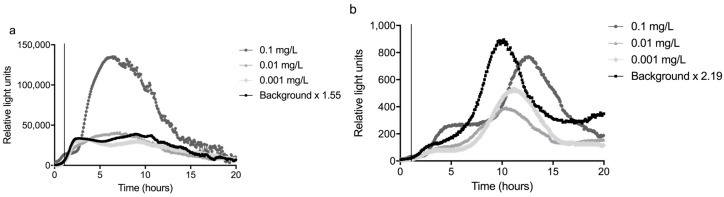
The result of spike experiments performed in the laboratory to determine the limit of detection of the sensor. The response of the sensor to different concentrations of mitomycin C is given, which were administered between 1 and 2 h (indicated by the vertical line), and the curve of the average background response x the response ratio of respectively 1.55 for the slide (**a**) and 2.19 for the fiber (**b**).

The sum of the average and three times the standard deviation of the background (n = 3) was divided by the average to calculate the response ratio. The highest response ratio between 2 and 10 hours was used as a significance limit; this was 1.55 for the glass slide and 2.19 for the fiber (Figure 4 and Figure 5). The LOD was defined as the lowest concentration that gave a response that was above this significance limit (i.e., 1.55 or 2.19 times the average background) in the time period between 2 and 10 h of monitoring time, as visualized in Figure 5.

The bacteria were exposed to several concentrations of mitomycin C, the most potent compound in the well plates (see Supplementary data, Part I), between 1 and 2 hours from the start of the experiment. The signal started to differ from the background about 1 hour after the start of the exposure to 0.1 mg/L mitomycin C, thus about 2 h from the start of the experiment. For lower concentrations, the response time became longer: up to three h for 0.01 mg/L mitomycin C, as can be seen in Figure 5. This shows a time frame of more than one hour is necessary between the passage of a contamination through the sensor system and the arrival of the contamination at the inlet for drinking water production, to be able to close the inlet. In other words, this sensor system would need to be placed at least one hour upstream of an inlet for drinking water production. For the concentrations of 0.01 mg/L and 0.001 mg/L, the averages of two experiments were plotted.

Using the definition above, the LOD for detecting mitomycin C was 0.01 mg/L for the glass slide setup and 0.1 mg/L for the fiber configuration of the sensor. The higher LOD of the fiber was mainly caused by the variation in the background peak of this setup. Especially the variation in the time at which this peak occurred reduced the sensitivity of the fiber. The variation on the slide was relatively low compared to the background, resulting in a higher sensitivity.

It is also noteworthy that the response to mitomycin C endured long after exposure to the inducing compound was stopped, especially after exposure to a concentration of 0.1 mg/L. HPLC analysis of samples from the sensor showed that no mitomycin C was detectable in the water 30 min after stopping the input of the toxicant. This is related to the fact that the bacteria are a living, multiplying system and DNA damage repair does not immediately stop after the genotoxic substance is removed. The implication for field monitoring is that once a positive signal has been induced, either the bacteria have to be replaced or a long lag period must be taken until the bacteria return to their normal state.

### 3.2. Evaluation Immobilization Methods

The bacteria were immobilized on both a glass slide and an optical fiber, which were used simultaneously to compare their relative performance. Fibers have been used in previous studies, thus they were already known as a viable platform for immobilization of bacteria and guidance of the light to a photodetector [22,25]. In this study, fibers were used with a larger diameter than previously, namely 1800 µm instead of 400 µm. The motivation behind this choice was that with a larger surface of the tip, more bacteria are directly in front of the fiber. Therefore, a larger portion of the emitted light is coupled into the fiber. It was also for this reason that glass slides were tested as immobilization platform, as they have a larger surface area than fibers. The idea was that by being able to gather more light, the resolution of the signal as well as the sensitivity would increase. 

Indeed, one can see the increased sensitivity in Figure 4 and Figure 5, the signal from the slide is often more than a factor hundred higher than that of the fiber. This is easily explained by the surface area differences (fiber-optic 5 mm^2^ and flat slide 1000 mm^2^) (Figure 1). 

More unexpected was the difference in shape of the background response from the bacteria. The bacteria on the slide always started with a steep rise in response, followed by a slow decrease after about 10 h. The response of the bacteria on the fiber increased more slowly and showed a characteristic peak between 10 and 14 h from the start of the experiment. This pattern remained when a mutagenic compound was added before the peak, although an increase in light production was observed one hour after the start of the exposure. In comparison, the response of the slide was sharper and probably easier to distinguish when the exact time of the dosing is not known. 

More importantly, the LOD of the slide was lower, namely 0.01 mg/L versus 0.1 mg/L for the fiber. This difference was caused by the large variation in the amplitude and time of the background peak of the fiber. An additional issue with the fiber was that the attachment of the gel was relatively weak and as a result, the gel was often lost during transport. As a result, only the glass slide was used in the field experiments.

### 3.3. Sensitivity of the Sensor in the Field

After determining the response of the sensor in the laboratory, the sensor was moved to the water monitoring station Keizersveer in Hank, The Netherlands. There, the sensor was tested with water from the river Meuse. The integration time of the PMT of the glass slide was set to 100 ms instead of the 10 ms used in the laboratory to reduce the noise of the signal. The bacteria were prepared in the laboratory and transported to Keizersveer by car. 

**Figure 6:**
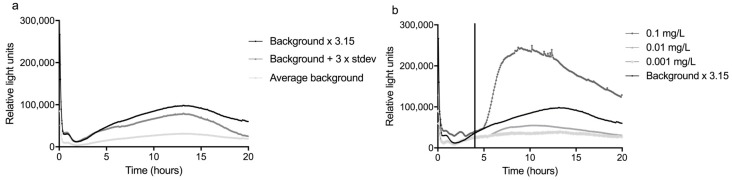
Determination of the background and response of the sensor at monitoring station Keizersveer. (**a**) shows the average of five background experiments, the average background + 3 times the standard deviation and the average background x the response ratio of 3.15. In (**b**), the response to three concentrations of mitomycin C, added between 4 and 5 h, are compared to the background x 3.15.

To reduce the amount of fouling, two filters were installed in front of the sensor. These filters were first tested with river water in the laboratory, as detailed in the next paragraph. Possibly as a result of the higher nutrient content of the Meuse water, a very strong rise in signal occurred after 10–17 h, which disrupted the measurements (Figure 6). This problem was solved by reducing the medium concentration by a factor 10 to 0.2% LB. 

The LOD was determined in the same way as for tap water, except that mitomycin C was added after 4 h from the start of the experiment to avoid an effect of the handling of the glass slide at the beginning of the experiments (Figure 6). In addition, the average and standard deviation of five background curves was used, instead of three. By taking the highest response ratio between 4 and 20 h, the response ratio when using Meuse water was 3.15. 

The lowest concentration that caused a response of more than 3.15 times the average background was defined as the LOD. In Figure 6, the response curves of three concentrations mitomycin C are shown, as well as the curve of the average background times 3.15. It can be seen that, although there was already a concentration-dependent increase in luminescence at lower concentrations, only 0.1 mg/L mitomycin C was sufficient to cause a significant response. Considering that the 0.1 mg/L concentration far exceeded the RR x background, it may well be that the LOD is actually somewhat lower, but concentrations between 0.01 and 0.1 mg/L were not tested. 

The higher LOD in river water compared to tap water (LOD of mitomycin C in tap water was 0.01 mg/L) can mainly be attributed to the higher variation in the background. This is not surprising, since the quality and composition of the Meuse water varies more than that of tap water. It is an important effect that should be kept in mind when determining the LOD of a sensor under field conditions. Unfortunately, the LOD as determined in this study is still too high to enable the detection of genotoxic compounds at the target value for these compounds in drinking water of 10 ng/L [26]. By reducing the variation in background between experiments, by for example standardization and/or automation of the measurements, it is expected that the sensitivity of the sensor will increase. Another option to increase the sensitivity might be pre-concentration of the water. It should be mentioned in this respect that also for *in vitro* genotoxicity assays, the water samples are usually concentrated to gain sufficient sensitivity [27]. However, this can probably not be combined with continuous on-line monitoring and instead would require a (semi-) batch like setup of the sensor. 

### 3.4 Fouling and Filtration

**Figure 7:**
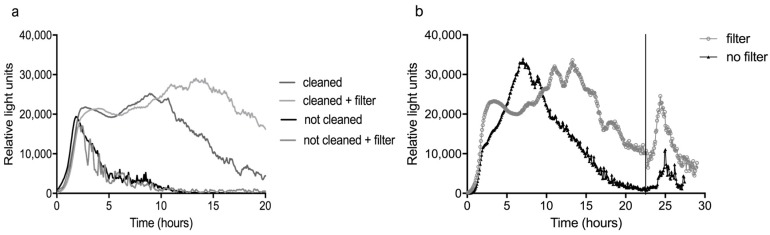
The effect of filtration on the response of the bacteria is shown. These experiments were performed with tap water in the laboratory. In (**a**) the background response is compared in both a cleaned sensor and when the sensor was used on subsequent days without cleaning in between and with and without the addition of a filter before the water inlet. The curves of the cleaned sensor are averages of three measurements, those of the not cleaned sensor single measurements. In (**b**) the bacteria were exposed to 1 mg/L nalidixic acid after 22.5 h, for one hour (moment indicated by the vertical line), with and without a filter.

Prior to every experiment, the sensor was cleaned with diluted bleach to remove biofouling from the system. In the laboratory we tried to perform experiments constitutively with tap water without cleaning in between. In these cases, the signal of the bacteria dropped to zero after three to five h and on the second day, no response to compounds could be seen (Figure 7). Culturing of water and biofilm samples from the sensor on LB agar plates showed that high numbers of contaminating bacteria, probably originating from the tap water, were present in the sensor when several experiments were run in succession without cleaning. Their numbers were particularly high from the point where LB broth was added to the water. Visual inspection confirmed the formation of biofilm in the Teflon tubing as well as on the sol-gel on the glass slide. Although only 2% medium was used, apparently the nutrient concentration was sufficiently high to accommodate a rapid growth of bacteria. This effect was probably enhanced by the temperature of 26 °C. It therefore seems that fast growing native bacteria in water may disrupt the signal of the reporter bacteria.

Previous reports of bacterial biosensors rarely discuss the occurrence of biofouling or its effect on the response of the bacteria [28]. The problem was usually avoided by using only sterile water [16,19] or by only performing short experiments with filtered field water [29].

Prior to the start of the field tests, a short study was performed to test the effectiveness of filtration in reducing biofouling and prolonging the response of the bacteria, and to determine the difference in response between tap and river water. Nalidixic acid was used as inducer in these and all further experiments, because this compound is much less toxic to higher organisms compared to mitomycin C. For more information on the response of the bacterial strain to nalidixic acid, see Supplementary data, Part I.

As can be seen in Figure 7a, the use of the two filters (0.5 µm and 30 nm) indeed resulted in a delay of the decline of the average background response (n = 3) in tap water. This was an improvement, because a decrease in background usually coincides with a decline of the responsiveness of the bacteria. Indeed, the response of the bacteria to 1 mg/L nalidixic acid, added after 22.5 h, was more pronounced when the filters were used (Figure 7b). Unfortunately, it was not possible to prevent biofouling sufficiently to enable experiments on subsequent days without cleaning in between, as the ‘not cleaned + filter’ curves clearly show. Since faster formation of fouling of the sensor was expected with surface water, the filters were used in all field experiments. 

This strong effect of fouling has strong practical implications for the use of this and similar sensors in the field, because it means they require daily maintenance. To enable monitoring over multiple days without cleaning and replacement of the bacteria would require a different setup of the sensor. Possible solutions may include further sample preparation, for example in the form of on-line Solid Phase Extraction (SPE), and/or some form of automatic replacement of the bacteria. 

### 3.5. Comparison with Other Monitors at Keizersveer

**Figure 8:**
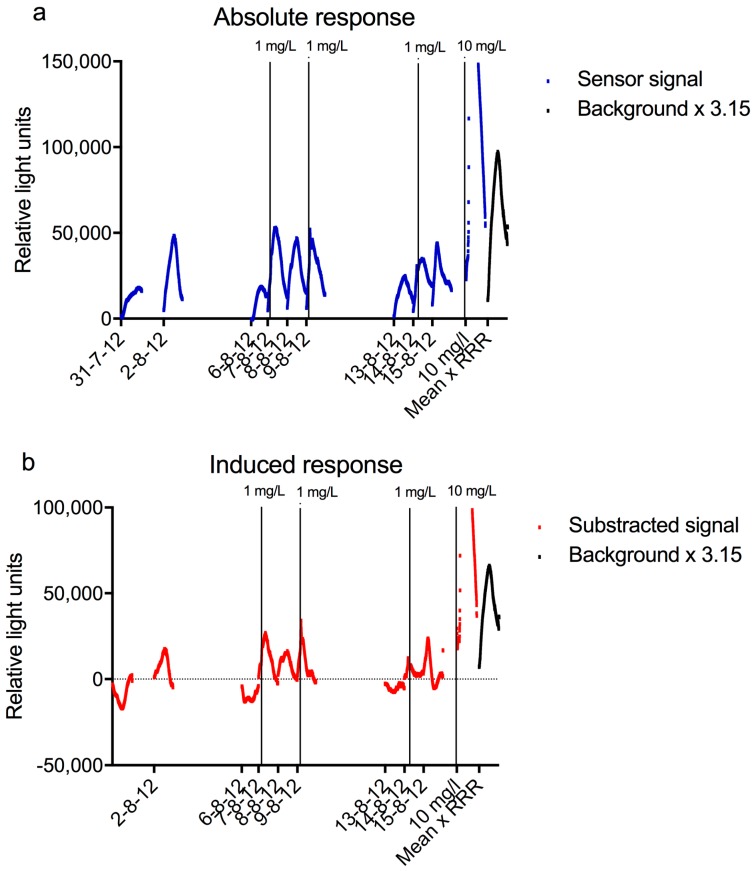

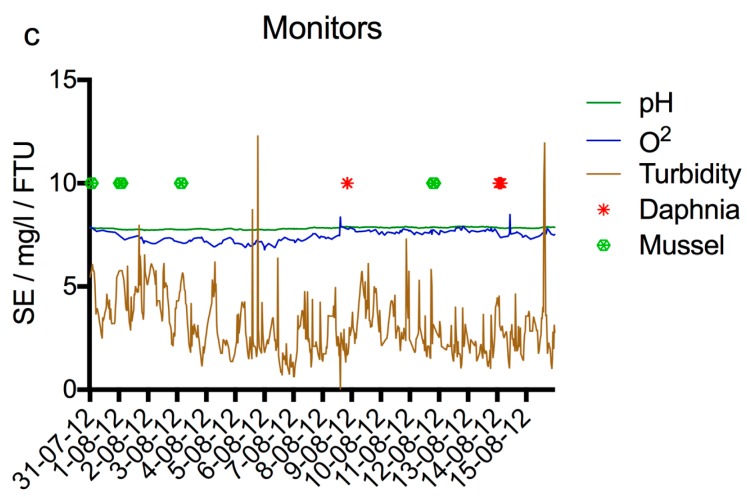
The response of the sensor, compared to other (bio)monitors at the Keizersveer monitoring station, Hank. For the Daphniatoximeter and the Musselmonitor only the alarms are shown. All bacterial sensor measurements were performed with 0.2% LB and continuous exposure to Meuse water. The vertical bars indicate when spikes of nalidixic acid were added to the water in the sensor. The duration of the spikes was always one hour. Graph (**a**) shows the unaltered signal of the sensor, while in graph (**b**) the average background was subtracted from the original response. The signals from the other (bio)monitors are shown in graph (**c**) (FTU: Formazine Turbidity Units, SE: standard pH units).

Several other (bio)sensors were also running at Keizersveer for regular water quality monitoring during the period the sensor was tested there. Among them were the *Daphnia*toximeter (bbe-Moldaenke, Schwentinental, Germany) and the Musselmonitor^®^ (AquaDect, Brouwershaven, The Netherlands) for on-line toxicity monitoring, and pH, temperature, oxygen, acidity and turbidity sensors. In Figure 8, the data of these monitors are given for the period between 31-07-2012 and 16-08-2012, as well as the response of the bacterial sensor during the same period and the response of the sensor after subtraction of the average background. All experiments with the bacterial sensor shown in this Figure were performed with 0.2% LB. The sensor was cleaned and new bacteria were inserted each day. Most of the experiments were done with unaltered Meuse water, except those of 7, 9, and 14 August 2012, when a spike of 1 mg/L nalidixic acid was dosed between 4 and 5 h. The curve of an experiment performed on 28 August 2012, when 10 mg/L nalidixic acid was added as a spike between 4 and 5 h, is shown as well. The last curve in Figure 8 shows the average x 3.15, which is the limit that should be passed to obtain a significant response. It can be derived from these curves that 10 mg/L nalidixic acid caused a very strong response in the sensor, while 1 mg/L failed to give a distinguishable increase in signal.

This graph clearly shows the typical daily pattern in the response of the sensor, with an increase in luminescence followed by a decrease. The increase is probably due to replication of the bacteria, while the decrease is thought to be caused by a combination of a reduction of the growth of the luminescent bacteria and fouling with other microorganisms. To prevent the reduction in signal and enable longer measurements would require a different setup of the sensor. One way to keep the bacteria at a constant density and growth phase is by keeping them in a continuous culture, as has been done with a few bioreactor sensors [15,16]. The disadvantages of these reactors however, include a relatively high dilution rate of the sample water and a high concentration of genetically modified bacteria in the wastewater. Another, more practical alternative, might be a (semi)batch design, with bacteria in wells plates and automated dosing. In such a system, new bacteria are used for every measurement [30]. The additional advantage is that a (semi)batch system can be coupled to on-line SPE to increase the sensitivity of the sensor.

The pH, oxygen concentration, and turbidity varied little during the test period of the sensor and no influence of these parameters was observed on the sensor signal. The *Daphnia*toximeter and the Musselmonitor^®^ both gave several alarms during the same time period, none of which coincided with an increase or decrease of the signal of the bacterial sensor beyond the normal variation. HPLC and GC-MS analysis showed that several industrial chemicals and pesticides were present in the low µg/L range on the days of the alarms, as well as unknown polar compounds detected using HPLC analysis (unpublished data). Since none of these measured compounds are known to have a strong genotoxic effect, it is not surprising that the bacterial sensor did not show an increase in luminescence above the background. 

Although the period during which the sensor was tested in the field was relatively short, based on the results obtained and the knowledge available of the compounds that occur in surface water, it is expected that a positive signal will not occur often. However, many water pollutants are not detected and identified with standard analytical methods. This is clearly shown by the alarms generated by the *Daphnia*toximeter and the Musselmonitor^®^, which are often caused by compounds that cannot be identified by standard HPLC analysis [9]. It should also be kept in mind that if there is a release of a genotoxic contaminant, an on-line sensor has a far higher probability of detecting such a release than routine sampling. As the experiments with nalidixic acid and mitomycin C spikes show, the sensor responded well to such sudden increases in genotoxic compounds in surface water.

## 4. Conclusions

A new biosensor was developed and tested for the on-line monitoring of genotoxic compounds in water using luminescent bacteria. In the sensor, the bacteria were immobilized on both the tip of an optical fiber and a glass slide. The light signal from the bacteria on the glass slide was much stronger than from the fiber, because of the larger surface area. Moreover, the variation between experiments was higher with the fiber, which reduced its sensitivity. The limit of detection of 0.1 mg/L mitomycin C was reached with the fiber and 0.01 mg/L mitomycin C with the glass slide. The time between the start of the exposure and the response was about one hour for both fiber and glass slide. Overall, the glass slide proved to be preferable over the fiber as immobilization method.

The sensor was also tested in a field monitoring station along the river Meuse. The sensor responded well to spikes of genotoxic compounds added to the river water, with a limit of detection of 0.1 mg/L mitomycin C for the glass slide. The *Daphnia*toximeter and Musselmonitor^®^, which were employed at the same station, generated several alarms during the test period, none of which resulted in a parallel alarm of the bacterial sensor. This was not unexpected, since these monitors respond to different compounds and toxic effects. Unfortunately, daily cleaning of the sensor was necessary, as fouling severely impaired the signal of the luminescent bacteria. 

This sensor was already a large improvement over its predecessor in stability, robustness and sensitivity. For future use of this on-line bacterial sensor in field studies, it is required to further improve the sensitivity (e.g., by reducing the variation between experiments) as well as the time needed for daily maintenance of the sensor. Both might be achieved by further standardization of the methods and simplification and/or automation of the sensor.

## Supplementary Materials

Supplementary materials are available online at : http://www.mdpi.com/1424-8220/17/11/2682/s1.
