# Supplementary material for: Development and Validation of an On-Line Water Toxicity Sensor with Immobilized Luminescent Bacteria for On-Line Surface Water Monitoring
Source: Sensors (Basel). 2017 Nov 22;17(11):2682. doi: 10.3390/s17112682 (PMC5713466; doi:10.3390/s17112682)
Supplement: Supplementary file 1 [file sensors-17-02682-s001.pdf]

Supplementary data

Part I: Response characterization of strain DPD2794

Chemicals

Ampicillin, mitomycin C, 4-nitroquinoline-N-oxide (4-NQO), cyclophosphamide, 2,4-diaminotoluene, p-chloroaniline, sodium saccharin, amitrole, p-nitrophenol, carbamazepine, nitrilotriacetic acid (NTA), tetrachloroethane, benzotriazole, and metham sodium were purchased from Sigma Aldrich (Zwijndrecht, The Netherlands).

Peptone, glycerol, lysogeny broth (also known as Luria-Bertani medium or LB), agar, dimethylsulfoxide (DMSO), nalidixic acid, cyclohexane, tertiary-butylhydroquinone (TBHQ), urea, di(2-ethylhexyl)phthalate (DEHP), methyl tert-butyl ether (MTBE), ethyl tert-butyl ether (ETBE), glyphosate, aminomethylphosphonic acid (AMPA), diuron, and atrazine were purchased from Boom (Meppel, The Netherlands).

Chloramphenicol was purchased from Fagron BV (Capelle aan de IJssel, The Netherlands). Iomeprol was purchased as a solution of 612.4 mg/mL from a local pharmacy and produced by ALTANA Pharma BV (Hoofddorp, The Netherlands). Dimethylnitrosamine-D6 (NDMA-D6: Deuterated NDMA was used since NDMA was not available) was purchased from Buchem BV (Apeldoorn, The Netherlands). All chemicals except iomeprol were of analytical grade and stored as suggested by the manufacturer's instructions.

Stock solutions of mitomycin C, nalidixic acid, 4-NQO, tetrachloroethane, diuron, p-chloroaniline, p-nitrophenol, cyclophosphamide, cyclohexane, MTBE, urea, chloramphenicol, ETBE, atrazine, TBHQ, carbamazepine, DEHP, amitrole, sodium saccharin, iomeprol, 2'4-diaminotoluene, NDMA-D6, and metham sodium were prepared in DMSO and stored at -80 °C.

Stock solutions of amitrole, NTA, benzotriazole, glyphosate, and AMPA were prepared in ultrapure water because of the poor solubility in DMSO and stored at 4-7 °C.

Bacteria and growth conditions

The genetically modified *E. coli* strain DPD2794 was obtained as a kind gift from R. Marks (Hebrew University, Jerusalem, Israel).

Stock cultures of bacteria were stored at -80 °C in 25% (v/v) glycerol medium. Colonies for daily use were maintained at 4 °C on LB-agar plates, supplemented with 100 mg/L ampicillin. Before each experiment, bacteria were grown overnight at 37 °C in an incubator (ETK combi, Elbanton, Kerkdriel, The Netherlands) on a rotary shaker (KS-500, Kika-werk) at 100 rpm in LB, which contained 100 mg/L ampicillin. The culture was then diluted to an OD_{600} of 0.2 as determined by a spectrophotometer (Jenway 6300 VIS, Staffordshire, England).

Experimental procedure exposure experiments

For all tested substances, concentration series were prepared of 9 to 14 concentrations per compound in DMSO. Before each experiment, the solutions were further diluted 25-fold by adding 4 µl of each concentration to 96 µl sterile tap water. The final concentrations can be found in Tables S1 and S2.

All experiments were performed in white 96-well plates (Nunc, Roskilde, Denmark). In each well, 25 µL diluted aqueous solution of the compounds was mixed with 25 µL LB and 50 µL bacterial culture, thus reaching a total dilution of a hundred times. When DMSO was used as solvent, all negative controls received a similar 1% DMSO. All concentrations were tested in triplicate. The tests with mitomycin C, 4-NQO, sodium saccharin, cyclophosphamide, glyphosate, carbamazepine, and ETBE were repeated on a different day as an independent control. The tests with nalidixic acid, chloramphenicol, and benzotriazole were repeated four times on different days. These repeats were used to determine the variation between different experiments for this assay.

Luminescence measurements were taken every 15 min for 23 h in a Luminoskan Ascent (Thermo Fisher Scientific, Breda, The Netherlands) plate luminometer. During the experiments the temperature was 26 °C and the plates were shaken with 60 rpm. The experiments were performed by 26 °C, because the luciferase enzyme becomes inactive above 30 °C.

<u>Response characterization of strain DPD2794 for various compounds in well plates</u>

The response of the genetically modified *E. coli* strain DPD2794 for the detection of DNA damage was determined for 27 compounds: 13 listed genotoxicity test validation compounds (Table S1) and 14 compounds detected regularly in surface waters (Table S2).

As can be seen in Figure S1, the bacteria always gave a background response, which was similar between experiments. For the determination of the limit of detection (LOD), the average values of the background curves of the compound series triplicates, which were repeated five times (n = 15), plus three times the standard deviation were plotted for each time point. Above these absolute values, a response can be seen as deviating significantly from the background. As the average and standard deviation varied in time, it was more convenient to convert these absolute limit values to ratio's and take the highest ratio as the worst case scenario.   Therefore, the resulting absolute limit value at each time point (average + 3*stdev of background) was then divided by the average value at the same time point, which resulted in a set of ratio's over time. Between two and 10 h (the timeframe where the genotoxic compounds produced a peak in luminescence, and thus the most relevant timeframe) the highest value for this ratio was 1.9.

The average of the responses to a sample (n = 3) was also divided by the average of the background measured in the same series (n = 3), at the time point where the difference between the background and exposed bacteria was the largest. Due to the different characteristics and concentrations of the compounds this point was not fixed, but always lied between two and ten h from the start of the experiment. The lowest concentration that induced a ratio of more than 1.9 was considered the limit of detection, because this was the lowest concentration of which the response was observed to deviate significantly from the variable background response.

In Figure S1, the results of nalidixic acid are shown as an example. Two h after the start of the exposure a concentration of 0.5 mg/L nalidixic acid induced a response more than 1.9 times the background signal whereas the response to a concentration of 0.1 mg/L nalidixic acid remained below this level throughout the whole measurement. Thus the limit of detection for nalidixic acid was determined to be 0.5 mg/L.

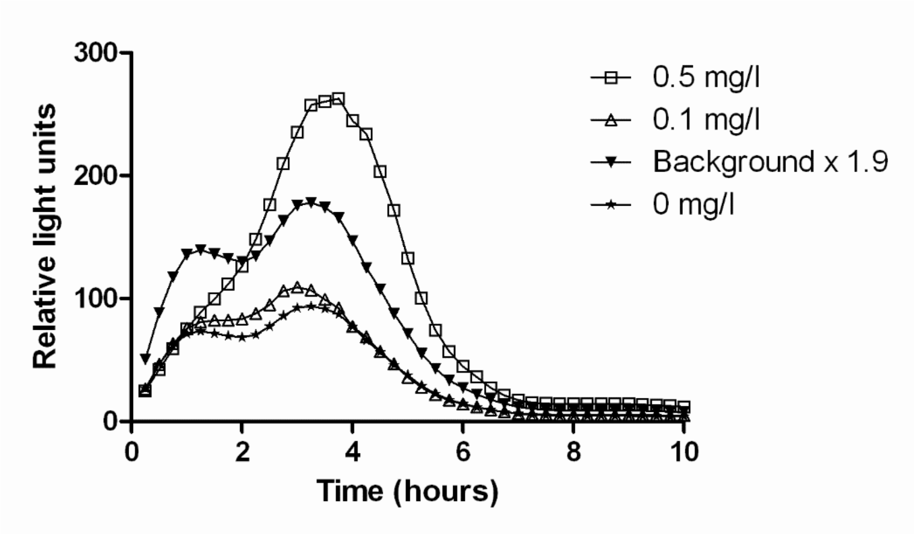

Figure S1. An example of the determination of the LOD for nalidixic acid. The concentration of 0.5 mg/L gave a response with a response ratio of more than 1.9 times the background, and was thus considered the detection limit for this compound.

Three of the 13 tested ECVAM-recommended chemicals gave a positive response, namely nalidixic acid, mitomycin C, and 4-NQO. The strain was most sensitive for mitomycin C, which already induced a response at a concentration of 0.0001 mg/L (Figure S2). This was in accordance with results reported for these compounds in the Ames mutagenicity assay, without metabolic activation. The genotoxic compounds that required metabolic activation, namely cyclophosphamide, 2,4-diaminotoluene and p-chloroaniline, did not induce a response in strain DPD2794. There was one false positive result for chloramphenicol, which was due to cytotoxicity, rather than a genuine genotoxic response. None of the non-genotoxic compounds induced a response in strain DPD2794.

None of the 14 environmental pollutants showed a positive response using strain DPD2794 in the microplate assay. This is in accordance with Ames test results reported in literature for several of these compounds. Although the mechanism behind the Ames test is different, it is a well-standardized test for gene mutations and can as such serve as a good indicator. A summary of the results is given in Tables S1 and S2.

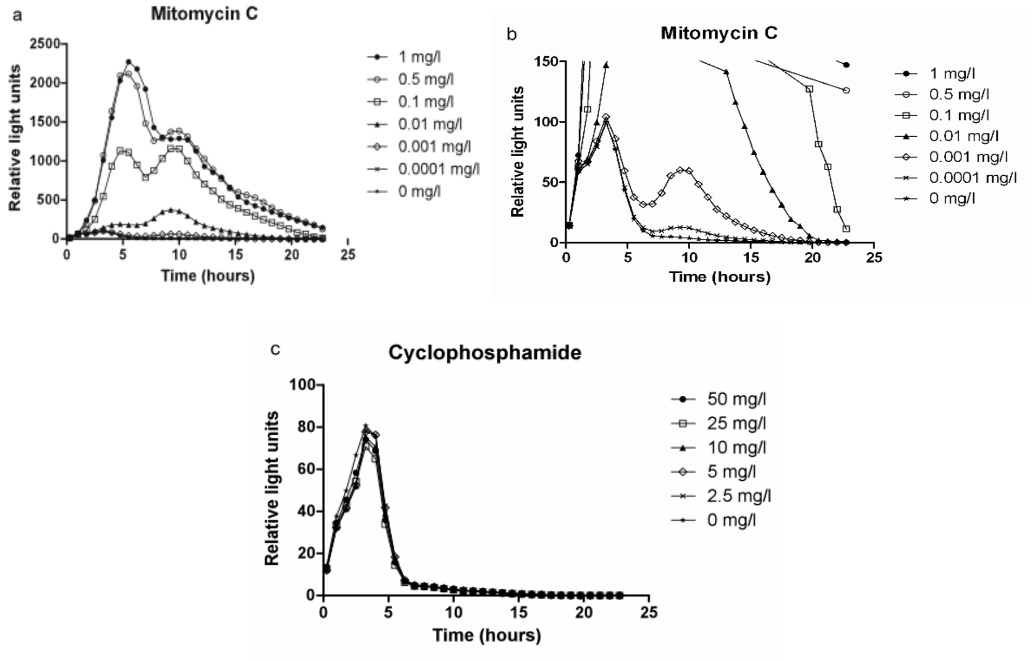

Figure S2. Average response of DPD2794 to positive and negative controls: (**a**) mitomycin C (n = 6), (**b**) an enlargement of the lower concentration curves of mitomycin C, (**c**) cyclophosphamide (n = 15). All experiments were performed over 23 h and the concentrations are the final concentrations in the wells.

Table S1. Overview of the results of the selected compounds recommended for validation of genotoxicity tests.

**Chemical**	**CAS nr.**	**DPD2794 response**	**LOD (mg/L)**	**Conc. range tested (mg/L)**	**Max absolute induced response** [1]	**Ames result [1]**	**IARC classification [2]**
nalidixic acid	389-08-02	+	0.5	0.001 - 10	1081	+	-
mitomycin C	50-07-07	+	0.0001	0.0001 - 1	2263	+	2B

4-NQO	56-57-5	+	0.5	0.001 - 10	814	+	-
cyclophosphamide	6055-19-2	-	-	1 - 50	-	+ (S9)[2]	1
chloramphenicol	56-75-7	+	0.75	0.05 - 10	76	-	2A
urea	57-13-6	-	-	0.1 - 1000	-	-	-
cyclohexane	110-82-5	-	-	1 - 100	-	-	-
TBHQ	1948-33-0	-	-	0.5 - 50	-	-	-
Sodium Saccharin	128-44-9	-	-	5 - 100	-	-	-
amitrole	61-82-5	-	-	5 - 100	-	-	3
2, 4 – diaminotoluene	95-80-7	-	-	0.5 - 200	-	+ (S9)[2]	2B
p-Nitrophenol	100-02-7	-	-	0.01 - 10	-	-	-
P-Chloroaniline	106-47-8	-	-	0.01 - 10	-	+ (S9)[2]	2B

1) The absolute highest difference between the response and the background
2) Metabolic activation was required for a response in the Ames test

Table S2. Overview of the results of the environmental compounds. ND = No Data

Chemical	CAS nr.	DPD2794 response	Conc. range tested (mg/L)	Use	Ames test result [1]	IARC classification [2]
carbamazepine	298-46-4	-	0.01 - 10	Drug	ND	-
iomeprol	78649-41-9	-	7.5 - 100	Drug	ND	-
DEHP	117-81-7	-	0.01 - 10	Plasticizer	-	-
MTBE	1634-04-4	-	7.5 - 100	Gasoline additive	-	3
ETBE	637-92-3	-	7.5 - 100	Gasoline additive	-	-
glyphosate	1071-83-6	-	0.01 - 10	Herbicide	-	-
AMPA	1066-51-9	-	0.001 - 10	Glyphosate metabolite	-	-
NTA	139-13-9	-	0.001 - 10	Chelating agent	-	2B

diuron	330-54-1	-	0.001 - 10	Herbicide	+ (S9)	-
atrazine	1912-24-9	-	0.01 - 50	Herbicide	-	3
benzotriazole	95-14-7	-	0.01 - 100	Corrosion inhibitor	+ (S9)	-
metham sodium	137-42-8	-	0.01 - 21	Fungicide	ND	-
NDMA-D6	17829-05-9	-	7.5 - 250	Industrial chemical	ND	-
tetrachloroethane	79-34-5	-	1 - 10	Solvent	ND	3

Part II Effect of LB medium concentration on the response

The first priority after the construction of the new sensor was to establish a stable and reliable signal. An essential factor in achieving this was adjustment of the concentration of nutrients available to the bacteria. In a previous study, it has already been shown that this strain of genetically modified *E. coli* did not respond without the addition of medium to the water [3]. The optimum concentration found in that study was 7.5% LB medium, which was used as starting point in the current study.

The response with 7.5% LB medium showed very strong fluctuations in luminescence after about 5 h in this sensor, both as background signal and after exposure of the bacteria to 0.1 mg/L mitomycin C. The reason for the strong fluctuations is not clear. These fluctuations were accompanied by a strong increase in background luminescence, which might have been caused by growth of the bacteria. We speculate that growth of these bacteria leads to luminescence as cell division includes DNA duplication, which includes DNA repair when DNA damage is detected. There always is some background DNA damage (i.e. spontaneous mutations) present in organisms, thus cell division will always lead to some DNA repair. In these modified bacteria, this will lead to some luminescence, even if no genotoxic substance is present in the water. Therefore, a high nutrient level, leading to increased growth of bacteria, could cause the observed increase in background luminescence.

For this reason, the LB concentration was reduced to two percent. As can be seen in Figure S3, a remarkable reduction of the fluctuations was observed at this concentration, without loss of the response on the slide. On the fiber, there was some reduction in response in the beginning of the curve, but the response was still clearly distinguishable. Also, the contrast between the background and the response levels increased as a result of a decrease in background luminescence. Only with the fiber a peak remained after about ten h. The cause of this peak remains unclear, especially since it did not occur on the slide. The observed lag-time, the time between the start of the dosing and the start of the response, remained the same as with 7.5% LB, namely about one hour. An additional advantage of a lower medium concentration was that it delayed the manifestation of biofouling in the system.

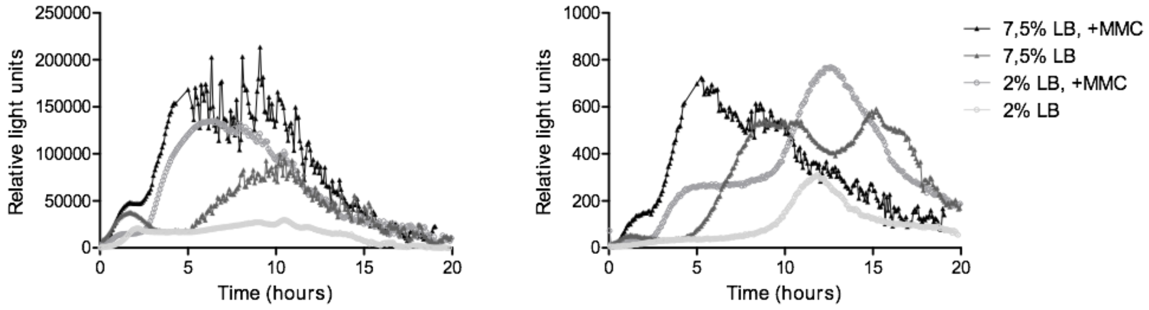

Figure S3. The effect of different nutrient concentrations on the response of the bacteria immobilized on a glass slide (left) or fiber (right). In two curves, mitomycin C was added between 1 and 2 h from the start of the experiment in a concentration of 0.1 mg/L.

Part III: Determination of the performance of UV-disinfection

The effectiveness of the disinfection was determined by collecting samples before and after the UV unit. The 0.1 mL samples taken before the UV-unit were diluted a hundred times, plated on LB agar plates and incubated overnight. Three 0.1 mL samples taken after the UV unit were directly plated on LB agar plates. One 100 mL sample taken after the UV unit was filtered over a 0.2 μm filter, which was also incubated on an agar plate.

<u>Evaluation of the UV-disinfection unit</u>

In several countries, including The Netherlands, the use of genetically modified bacteria is bound to strict Governmental or European regulations. This means that all contaminated material, including the waste water from the sensor, had to be disinfected. The use of chlorine bleach has been established as a valid method for the disinfection of water contaminated with genetically modified *E. coli* [3]. However, this method has the disadvantages that it cannot be used on-line and that the use and disposal of large quantities of chlorine is undesirable. Therefore, UV-C irradiation was tested as an alternative method of disinfection.

The collected agar plate counts from three separate laboratory experiments showed an average concentration of $9.4*10^4$ bacteria per mL in the waste water of the sensor at the end of a normal one-day experiment. No colonies were found on any of the agar plates with the samples taken after installation and activation of the UV unit, including that of the filtered 100-mL sample. This indicated that the UV unit was very effective at the inactivation of the bacteria (>log 6 removal), under laboratory conditions.

Unfortunately, during the field experiments there were still some colonies of undefined bacteria found after the UV-lamp, although their numbers were drastically reduced. Therefore, in addition to the UV-unit, bleach was still added to the water to be able to ensure a complete disinfection. In conclusion, the use of UV for disinfection showed promising results, but more research is needed to determine the light intensity and contact time required to ensure a complete inactivation of all bacteria under field conditions.

References

1. U.S. National Library of Medicine. Chemical carcinogenesis research information system (ccris). http://toxnet.nlm.nih.gov/cgi-bin/sis/htmlgen?CCRIS
2. IARC. Iarc monographs. http://monographs.iarc.fr/ENG/Classification/index.php
3. Eltzov, E.; Marks, R.S.; Voost, S.; Wullings, B.A.; Heringa, M.B. Flow-through real time bacterial biosensor for toxic compounds in water. *Sensors and Actuators B: Chemical* **2009**, *142*, 11-18.
